# Supplementary material for: Acute Kidney Injury in Patients Undergoing Total Hip Arthroplasty: A Systematic Review and Meta-Analysis
Source: J Clin Med. 2019 Jan 9;8(1):66. doi: 10.3390/jcm8010066 (PMC6352044; doi:10.3390/jcm8010066)
Supplement: Supplementary file 1 [file jcm-08-00066-s001.pdf]

Online Supplementary Data 1. Search terms for systematic review.

Databases: Ovid MEDLINE (205 articles)

1. exp acute kidney injury/
2. acute kidney injury.mp
3. AKI.mp
4. renal failure.mp
5. kidney failure.mp
6. 1 or 2 or 3 or 4 or 5
7. hip.mp
8. replacement.mp
9. arthroplasty.mp
10. 8 or 9
11. 7 and 10
12. 6 and 11

Databases: EMBASE: (205 articles)

('hip arthroplasty' OR 'hip surgery') AND 'acute kidney failure'

Database: Cochrane Databases

Search all text "acute kidney injury" AND " hip arthroplasty"

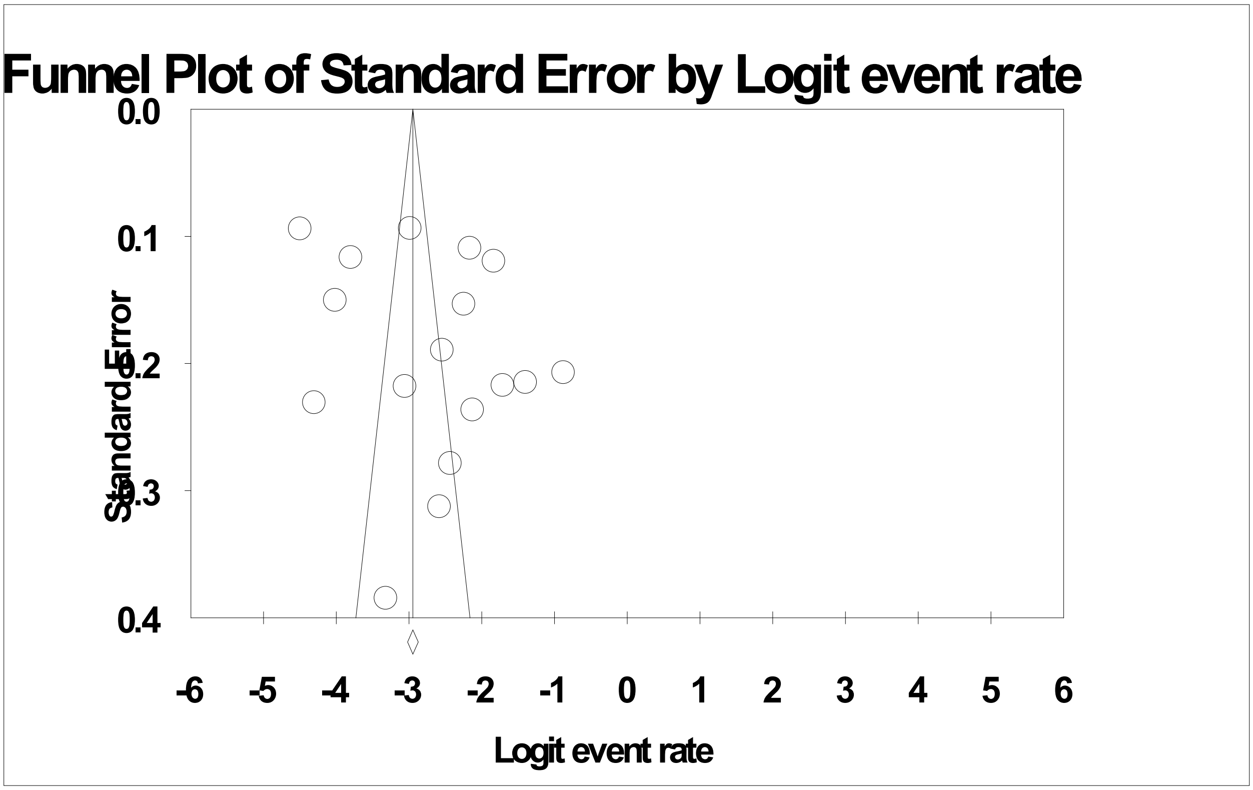

Supplementary Figure S1. Funnel plot evaluating for publication bias evaluating incidence of AKI in patients undergoing THA.
